# Supplementary material for: SS-31: A promising therapeutic agent against bleomycin-induced pulmonary fibrosis in Mice
Source: PLoS One. 2025 Apr 29;20(4):e0315473. doi: 10.1371/journal.pone.0315473 (PMC12040141; doi:10.1371/journal.pone.0315473)
Supplement: Attachment — Submitted filename: Response to Reviewers.pdf [file pone.0315473.s002.pdf]

Dear editor and reviewers

Thanks for revising this study. Our research team fully discussed and revised these comments again, and responded point-by-point to the review comments. Some revisions have been shown yellow in the manuscript.

Here is the point-to-point response

1. Please ensure that your manuscript meets PLOS ONE's style requirements, including those for file naming.

Response: We have revised our manuscript format according to your request.

2. To comply with PLOS ONE submissions requirements, in your Methods section, please provide additional information regarding the experiments involving animals and ensure you have included details on (1) methods of sacrifice, (2) methods of anesthesia and/or analgesia, and (3) efforts to alleviate suffering.

Response: We have added this to the manuscript in part 2.2.

3. Thank you for stating the following financial disclosure:

“This research was supported by Heilongjiang Province Key R&D Program(GA21C011) and the National Natural Scientific Foundation of China [82172164] and Heilongjiang Province Key R&D Program(JD22C005).”

Please state what role the funders took in the study. If the funders had no role, please state: ""The funders had no role in study design, data collection and analysis, decision to publish, or preparation of the manuscript.""

If this statement is not correct you must amend it as needed.

Please include this amended Role of Funder statement in your cover letter; we

will change the online submission form on your behalf.

Response: We have modified this section according to your opinion.

4. We note that your Data Availability Statement is currently as follows: All relevant data are within the manuscript and its Supporting Information files.

Please confirm at this time whether or not your submission contains all raw data required to replicate the results of your study. Authors must share the "minimal data set" for their submission. PLOS defines the minimal data set to consist of the data required to replicate all study findings reported in the article, as well as related metadata and methods (https://journals.plos.org/plosone/s/data-availability#loc-minimal-data-set-definition).

For example, authors should submit the following data:

- The values behind the means, standard deviations and other measures reported;

- The values used to build graphs;

- The points extracted from images for analysis.

Authors do not need to submit their entire data set if only a portion of the data was used in the reported study.

If your submission does not contain these data, please either upload them as Supporting Information files or deposit them to a stable, public repository and provide us with the relevant URLs, DOIs, or accession numbers. For a list of recommended repositories, please see https://journals.plos.org/plosone/s/recommended-repositories.

If there are ethical or legal restrictions on sharing a de-identified data set, please explain them in detail (e.g., data contain potentially sensitive information, data are owned by a third-party organization, etc.) and who has imposed them (e.g., an ethics committee). Please also provide contact information for a data access committee, ethics committee, or other institutional body to which data requests may be sent. If data are owned by a third party, please indicate how others may request data access.

Response: We have uploaded the experimental data at your request, named “minimal data set”

5. When completing the data availability statement of the submission form, you indicated that you will make your data available on acceptance. We strongly recommend all authors decide on a data sharing plan before acceptance, as the process can be lengthy and hold up publication timelines. Please note that, though access restrictions are acceptable now, your entire data will need to be made freely accessible if your manuscript is accepted for publication. This policy applies to all data except where public deposition would breach compliance with the protocol approved by your research ethics board. If you are unable to adhere to our open data policy, please kindly revise your statement to explain your reasoning and we will seek the editor's input on an exemption. Please be assured that, once you have provided your new statement, the assessment of your exemption will not hold up the peer review process.

Response: We have uploaded the experimental data at your request.

6. PLOS requires an ORCID iD for the corresponding author in Editorial Manager on papers submitted after December 6th, 2016. Please ensure that you have an ORCID iD and that it is validated in Editorial Manager. To do this, go to ‘Update my Information’ (in the upper left-hand corner of the main menu),

and click on the Fetch/Validate link next to the ORCID field. This will take you to the ORCID site and allow you to create a new iD or authenticate a pre-existing iD in Editorial Manager.

Response: I have added my ORCID iD to my Information at your request.

7. Thank you for stating the following in the Acknowledgments Section of your manuscript:

“This research was supported by Heilongjiang Province Key R&D Program(GA21C011) and the National Natural Scientific Foundation of China [82172164] and Heilongjiang Province Key R&D Program(JD22C005).”

We note that you have provided funding information that is not currently declared in your Funding Statement. However, funding information should not appear in the Acknowledgments section or other areas of your manuscript. We will only publish funding information present in the Funding Statement section of the online submission form.

Please remove any funding-related text from the manuscript and let us know how you would like to update your Funding Statement. Currently, your Funding Statement reads as follows:

“This research was supported by Heilongjiang Province Key R&D Program(GA21C011) and the National Natural Scientific Foundation of China [82172164] and Heilongjiang Province Key R&D Program(JD22C005).”

Please include your amended statements within your cover letter; we will change the online submission form on your behalf.

Response: We have modified this section according to your opinion.

8. PLOS ONE now requires that authors provide the original uncropped and unadjusted images underlying all blot or gel results reported in a submission’s

figures or Supporting Information files. This policy and the journal's other requirements for blot/gel reporting and figure preparation are described in detail at https://journals.plos.org/plosone/s/figures#loc-blot-and-gel-reporting-requirements and https://journals.plos.org/plosone/s/figures#loc-preparing-figures-from-image-files. When you submit your revised manuscript, please ensure that your figures adhere fully to these guidelines and provide the original underlying images for all blot or gel data reported in your submission. See the following link for instructions on providing the original image data: https://journals.plos.org/plosone/s/figures#loc-original-images-for-blots-and-gels.

In your cover letter, please note whether your blot/gel image data are in Supporting Information or posted at a public data repository, provide the repository URL if relevant, and provide specific details as to which raw blot/gel images, if any, are not available. Email us at plosone@plos.org if you have any questions.

Response: We have uploaded the original uncropped and unadjusted images underlying all blot results as a Supporting Information files named "WB".

9. Please review your reference list to ensure that it is complete and correct. If you have cited papers that have been retracted, please include the rationale for doing so in the manuscript text, or remove these references and replace them with relevant current references. Any changes to the reference list should be mentioned in the rebuttal letter that accompanies your revised manuscript. If you need to cite a retracted article, indicate the article's retracted status in the References list and also include a citation and full reference for the retraction notice.

Response: We have reexamined this section at your request.

Additional Editor Comments:

Dear Authors

Although this study is intriguing, a few issues need to be fixed before it can be accepted. Please include the study's goal at the conclusion of the introduction. Kindly add the euthanasia technique to the list of procedures.

Response: We have modified this section according to your opinion.

Reviewer #1: Review Report:

The manuscript titled "SS-31 protects against bleomycin-induced lung injury and fibrosis" suggests that the research focuses on the therapeutic potential of SS-31 in preventing or alleviating lung damage and scarring caused by bleomycin in experimental models.

Note: I reviewed the file named (PONE-D-24-54571) downloaded from journal online portal and all my comments are according to the line numberings of the pdf file “PONE-D-24-542471”

Detailed Comments/Recommendations:

Title:

The title of the study, "SS-31 protects against bleomycin-induced lung injury and fibrosis" effectively communicates the focus of the study, specifically the therapeutic role of SS-31 in addressing bleomycin-induced lung injury and fibrosis. appears to be apt and engaging. It reflects the experimental context and potential outcomes, making it relevant to researchers interested in mitochondrial-targeted therapies or pulmonary fibrosis models.

The title is suitable for the scope of the study, but slight refinements, such as specifying the experimental model or incorporating mechanistic details, would enhance its clarity, relevance, and appeal to the target audience.

Suggested Title: SS-31: A Promising Therapeutic Agent Against Bleomycin-Induced Pulmonary Fibrosis in Mice"

Response: We have revised the study title according to your recommendation

Abstract:

The abstract clearly states the purpose of the research, focusing on the potential protective effects of SS-31 against bleomycin-induced pulmonary fibrosis in mice.

Lines 57-59: SS-31 was administered daily but does not specify the dosage or route of administration. Including this detail would strengthen the methodological clarity.

Response: We have added the SS-31 administration method and measurement according to your comments

Lines 62-63: The study demonstrated that SS-31 could potentially mitigate the reduction in mice" is incomplete, leaving the reader unclear about what specific reduction is being referred to.

Response: We have revised the manuscript: BLM can cause a significant decrease in body weight in mice. However, intraperitoneal injection of SS-31 slowed down the body weight loss in mice.

Lines 62-70: While methods such as HE staining, Masson staining, and Western blot are mentioned, the abstract lacks numerical or statistical details to quantify the findings. Providing key metrics (e.g., percentage reduction in fibrosis or ROS levels) would enhance credibility.

Response: The specific results have been shown in the results, for simplicity, we do not specify in the abstract.

Lines 71-75: While the abstract mentions outcomes like reduced ROS and inflammatory factors, it does not delve into the specific mechanisms through which SS-31 exerts these effects. A brief mention of the proposed pathways would be helpful.

Address any typographical errors and ensure proper scientific notation.

Response: Regarding the mechanism of action of SS-31, we have shown in the discussion that, for simplicity, we did not specify it in the abstract.

We have re-checked the manuscript at your request to standardize the scientific notation.

Key Words: Three out of five key words are already mentioned in title. It will be more attractive if the writer mentions some other relevant key words.

Response: We have added the keyword "mitochondria" according to your suggestion.

Introduction:

Combine the 1st and 2nd paragraphs to provide a concise yet comprehensive summary of pulmonary fibrosis and its challenges.

In 2nd Paragraph: The section on pulmonary fibrosis repeats some information (e.g., limited treatments and poor prognosis). Streamlining these details can make the introduction more concise.

Response: We have modified this section according to your suggestion.

In 2nd paragraph: While the role of oxidative stress and ROS in pulmonary fibrosis is mentioned, the discussion could benefit from a brief elaboration on how SS-31 specifically counteracts these processes.

Response: We have modified this section according to your suggestion.

Lines 107 to onward: Include a specific mention of how SS-31 reduces ROS or preserves mitochondrial integrity, tying it directly to pulmonary fibrosis pathogenesis.

Response: We have modified this section according to your suggestion.

Lines 122 to onward: Clarify the connection between pulmonary hypertension and fibrosis, or reduce the emphasis on PH if it is not a major focus of the research.

Response: We have removed this part based on your recommendation.

Some references (e.g., oxidative stress role, SS-31’s effects) are summarized without detailed explanations or direct linkage to the study’s aims. Consider elaborating on key studies to provide stronger support for SS-31's potential.

Response: We have removed this part based on your recommendation.

Materials and Methods:

Lines 139-141: “The killing methods and model establishment methods

have passed the ethical standards...": Rephrase to improve readability and remove redundancy.

Response: We have modified this section according to your suggestion.

Lines 151-153: Terms like "molding" (e.g., "from 1 day before molding until day 28 after molding") are unusual and may confuse readers. Use "model induction" or "bleomycin treatment" instead.

Response: We have modified this section according to your suggestion.

Lines 162, Histopathology: The paraffin embedding and sectioning steps should clarify equipment or specific dehydration/embedding protocols.

Response: We have modified this section according to your suggestion.

Lines 192-193: ATP content in mouse lung homogenate was determined according to commercial protocols" can be rephrased as: "ATP levels in lung homogenates were measured using a commercial assay (Beyotime, China) following the manufacturer's instructions."

Response: We have modified this section according to your suggestion.

Lines 206 Western Blotting: While antibody details are comprehensive, the section lacks information about the imaging system or software used for quantification.

Response: We have modified this section according to your suggestion.

Line 234 RT-PCR: Mention the specific thermal cycling conditions used for amplification to enhance reproducibility.

Response: We have modified this section according to your suggestion.

Results:

Well presented.

Discussion:

The discussion thoughtfully contextualizes the findings within the broader literature.

It integrates findings with existing literature, particularly the role of oxidative stress, mitochondrial dysfunction, and the antioxidant potential of SS-31. However, the linkage between the experimental results and prior studies can

be elaborated further to highlight how this work advances knowledge in the field.

Response: We have modified this section according to your suggestion.

Incorporate more detailed comparisons between the experimental results and established models of pulmonary fibrosis to contextualize the significance of the observed improvements with SS-31.

Response: We have modified this section according to your suggestion.

While weight changes, histological evidence, and biochemical markers are valuable, a stronger emphasis on the potential for SS-31 to improve lung function (even if speculative) would be impactful.

Response: Thanks for your suggestion, this suggestion is very constructive.We also included this part in the limitations of our discussion section. However, we could not directly detect the lung function of C57BL /6 mice. It can only be proved indirectly by histology and by biological indicators. In future experiments, we will consider using rats or larger experimental animals, at which time we can directly test the effect of SS-31 on lung function.

Although the MAPK pathway is mentioned, additional exploration or hypothesizing about other mechanisms (e.g., ROS detoxification efficiency or mitochondrial biogenesis) would add depth to the discussion.

Response: We have modified this section according to your suggestion.

Consider simplifying some sections to improve readability, especially for readers less familiar with technical details. For instance, the paragraph on mitochondrial dysfunction could benefit from a summary sentence that ties findings to their clinical relevance.

Response: We have modified this section according to your suggestion.

Conclusion:

This is well written however future studies could benefit from a deeper dive into signaling pathways (e.g., MAPK) to uncover how SS-31 exerts its effects at the molecular level.

Response: We have modified this section according to your suggestion.

References:

Well documented

Concluding Remarks:

The paper necessitates the aforementioned corrections, inclusive of rectifying any errors highlighted, coupled with a meticulous proofreading by a proficient language expert to refine its English language usage. Upon addressing these amendments, the paper will meet the required standards for acceptance.

Reviewer #2:

The manuscript is well written. The purpose, methods and results are clearly presented.Studies involved with the live animals were carried out following protocols approved by the Research Ethics Committee. The statistical analysis in this paper is well conducted.The authors should report some of the limitations of this study in detailed.

Response: We have modified this section according to your suggestion.

Finally, thank you again for your valuable suggestions for this article. These amendments brought our research to a higher level, and we benefited a lot from this revision.

Sincerely yours,

Mingyan Zhao
